# Editorial: The editor's challenge: Cognitive resources

**DOI:** 10.3389/fpsyg.2022.994801

**Published:** 2022-09-01

**Authors:** Bernhard Hommel, Gesine Dreisbach

**Affiliations:** ^1^Department of Biopsychology, Technical University Dresden, Dresden, Germany; ^2^Institute of Psychology, University of Regensburg, Regensburg, Germany

**Keywords:** cognitive resource, executive control, cognitive control, attention, cognition

Many empirical and theoretical approaches in the cognitive sciences/neurosciences rely on the concept of *cognitive resources*. Selective attention and dual-task interference have been “explained” by resource limitations, thinking styles rely on the assumption that some cognitive processes are more resource-demanding than others, information integration is assumed to require precious cognitive resources, and so forth and so on.

And yet, no one knows what this resource is. Is it just a metaphor for something that we do not and can never really understand, or are we able to reveal its functional and/or neural basis? Is it just a shorthand for an emerging property of the dynamics of cognitive/neural processes and/or the interactions between competitive representations? How does that work, how do interactions deplete resources? Or does it really refer to some measurable “stuff” that is limited, like the amount of crosstalk/conflict between representations, sugar in the brain, dopamine, frequencies available for neural oscillations, or blood/energy? How can we measure this stuff, change its availability or dynamics? A truly mechanistic theory should offer testable assumptions about the structures/representations that are involved in embodying or generating resources and resource limitations, about the processes operating on these representations, and present a scenario explaining how the interactions between structure and process generate both resources and shortages thereof (Hommel, 2020)—at a level of detail that is open to empirical test and computational simulation.

Such a scenario is unlikely to be developed overnight, but we aimed to start this endeavor by inviting critical, ambitious, and courageous contributions of any kind, whether theoretical, conceptual, empirical, or computational, that provide important constraints for a better, truly mechanistic understanding of human cognitive resources. What are these resources, what do they stand for, where do they come from? We encouraged authors to throw all the homunculi out and take the next step, ideally in a broad, constructive discussion that transcends common communication bubbles.

Eleven authors or teams accepted our challenge. Two Systematic Reviews focused on working memory and attention, two areas in which resources play a particularly dominant role. Schumann et al. address a particularly modern topic: the relationship between multitasking and wellbeing. In particular, they are asking what experimental rest-break research is telling us about cognitive resources. They provide a taxonomy of rest breaks according to which empirical studies can be classified and then evaluate the theorizing in various fields, with an eye for popular concepts like ego depletion and opportunity costs. They distinguish between resource-based and satiation-based theoretical approaches and provide a set of guidelines for both theory building and future empirical approaches to the experimental study of rest breaks.

Vartanian et al. consider another obvious limitation of human information processing: working-memory (WM) span. While this span is often considered to be a structural limitation of the WM system, there is increasing evidence that WM capacity can be increased through individual training. The authors are asking whether training can change the neural substrates underlying WM and, if so, which systems are affected. Their meta-analysis of fMRI studies using WM training provides evidence suggesting that training is mainly targeting clusters within the fronto-parietal system, including the bilateral inferior parietal lobule (BA 39/40), middle (BA 9) and superior (BA 6) frontal gyri, and medial frontal gyrus bordering on the cingulate gyrus (BA 8/32). They discuss the functional and neural implications of these observations, as well as the implications for the construct of WM span as a limited resource.

In their Mini Review, Tagliabue and Mazza consider another limitation that will be affecting all of us sooner or later: the reduction of cognitive resources with increasing age. Age is assumed to be associated with a reduction of such resources, so that older individuals exhaust their resources more easily and more rapidly with difficult tasks. However, the authors emphasize that the most recent studies on neurophysiological markers of age-related changes are not overly consistent with respect to the relationship between neural and behavioral effects, which in turn suggests that neural indices may not be sufficiently diagnostic with respect to cognitive deficits. The authors further discuss possible confounds that might be responsible for the inconsistent picture and suggest possible ways to control them. They also suggest a theoretical alternative that considers age-related effects as qualitative, rather than quantitative, changes in the way cognitive resources are deployed at higher age.

In their Hypothesis and Theory article, Ansorge et al. compare traditional resource-limitation approaches to selectivity in human information processing to a functional approach that has a closer look at the necessities of information processing. The authors review various findings that have been taken to support the resource-limitation view, but point out that other interpretations are possible, sometimes even more plausible. Even apparent demonstrations of what looks like automatic processing, they argue, might be better understood from a functional point of view, and the same holds for what looks like neurophysiological evidence for resource limitations.

In the other Hypothesis and Theory article, Butz considers the nature of cognitive effort from a computational point of view. He suggests that a Bayesian brain approach has various advantages. The author describes how cognitive effort might be formalized in such an approach, and he develops a resourceful event-predictive inference model (REPI) that can successfully simulate effortful behavior. He discusses how the structure of this model accounts for interference effects, like in a Simon task, or for Task-switching costs. The further implications of the model are also considered.

In his Perspective article, Kleinsorge attributes the theoretical problems of the concept of cognitive capacity to Cognitive Psychology's failure to properly define the concept of representation in general and of task representation in particular. He emphasizes the central role of task instructions and describes how particular instructions can implement particular task spaces, as it were, the characteristics of which then generate what looks like capacity limitations as a side effect. He points out that a better understanding of these and related theoretical problems requires more research on instructions and how they shape the cognitive implementation of tasks.

In their Opinion article, Naefgen and Gaschler point out that cognitive research has tended to neglect variability of performance within individuals, and they argue that a stronger focus on this kind of variability might help us to understand the concept of cognitive resources in more depth. They present a method that allows distinguishing between cognitive resources and what they call common factors by using within-individual covariance patterns. They argue that resource limitations and common factors generate different data patterns, which they take as an important first step toward more mechanistic theorizing.

The Brief Research Report of Gallo et al. highlights the role of bilingualism in the development of cognitive resources and cognitive reserve. In their study, bilingual healthy seniors performed an online study, in which moderators of cognitive resource and second-language use were assessed. Structural Equation Modeling revealed facilitatory effects of L2 age of acquisition and L2 proficiency on executive performance and provided evidence for a moderating role of bilingual experience on the relationship between other factors known to promote cognitive reserve and cognitive integrity. Hence, bilingualism seems to play an important role in mitigating cognitive decline and promoting successful aging.

Three Original Research articles round up the Research Topic. Velasquez et al. focus on the conditions under which task-irrelevant stimuli can trigger involuntary conscious imagery. The authors presented their participants with video footage of events that one would observe from the driver's seat of a semi-automated vehicle, after having trained participants in such a way that street signs would be likely to induce involuntary imagery. Participants reported spontaneous involuntary imagery even if they were asked not to respond to the street signs and even under dual-task conditions. This suggests that imagery does not seem to underlie resource limitations.

Wang et al. are asking when and under which circumstances the completion of a task leads to the replenishment of cognitive resources. They show that the amount of replenishment depends on the current availability of resources and the cost-benefit trade-off at task completion. These observations provide further evidence for how people manage the investment of cognitive resources.

Finally, Senoussi et al. consider whether memory limitations reflect structural limitations of cognitive resources or a useful feature of human information processing. The authors suggest that flexible cognition requires time-based binding, which in turn necessarily limits the number of bound events that can be stored simultaneously. They believe that time-based binding is likely to be instantiated *via* neural oscillations and discuss supporting evidence.

Taken altogether, various avenues to specify, perhaps even to overcome the cognitive-resource concept exist, and the contributions to this Research Topic have suggested various theoretical, methodological and/or computational tools to make progress with respect to our understanding of the concept and its functional and neural underpinnings. Increasing doubts in the structural nature of possible resource limitations are obvious in many of the contributions, and various efforts to develop alternative interpretations have been made. As anticipated, these are only first steps and much more theoretical and empirical research will be necessary to really understand what the concept of resources is buying us, which theoretical alternatives are realistic and empirically supported, and whether it makes sense to replace the concept by more mechanistic descriptions. The interest in these questions seems to be widespread, as witnessed by the very substantial download rates for all 11 contributions. Hence, we are optimistic that research on this topic is moving forward.

## Author contributions

All authors listed have made a substantial, direct, and intellectual contribution to the work and approved it for publication.

## Conflict of interest

The authors declare that the research was conducted in the absence of any commercial or financial relationships that could be construed as a potential conflict of interest.

## Publisher's note

All claims expressed in this article are solely those of the authors and do not necessarily represent those of their affiliated organizations, or those of the publisher, the editors and the reviewers. Any product that may be evaluated in this article, or claim that may be made by its manufacturer, is not guaranteed or endorsed by the publisher.
